# Reducing disease burden and health inequalities arising from chronic disease among Indigenous children: an early childhood caries intervention

**DOI:** 10.1186/1471-2458-12-323

**Published:** 2012-05-02

**Authors:** Jessica Merrick, Alwin Chong, Eleanor Parker, Kaye Roberts-Thomson, Gary Misan, John Spencer, John Broughton, Herenia Lawrence, Lisa Jamieson

**Affiliations:** 1 Australian Research Centre for Population Oral Health, University of Adelaide School of Dentistry, Adelaide, Australia; 2 Menzies School of Health Research, Charles Darwin University, Darwin, Australia; 3 University of South Australia, Adelaide, Australia; 4 Ngai Tahu Maori Health Research Unity, University of Otago, Dunedin, New Zealand; 5 School of Dentistry, University of Toronto, Toronto, Canada

## Abstract

**Background:**

This study seeks to determine if implementing a culturally-appropriate early childhood caries (ECC) intervention reduces dental disease burden and oral health inequalities among Indigenous children living in South Australia, Australia.

**Methods/Design:**

This paper describes the study protocol for a randomised controlled trial conducted among Indigenous children living in South Australia with an anticipated sample of 400. The ECC intervention consists of four components: (1) provision of dental care; (2) fluoride varnish application to the teeth of children; (3) motivational interviewing and (4) anticipatory guidance. Participants are randomly assigned to two intervention groups, immediate (n = 200) or delayed (n = 200). Provision of dental care (1) occurs during pregnancy in the immediate intervention group or when children are 24-months in the delayed intervention group. Interventions (2), (3) and (4) occur when children are 6-, 12- and 18-months in the immediate intervention group or 24-, 30- and 36-months in the delayed intervention group. Hence, all participants receive the ECC intervention, though it is delayed 24 months for participants who are randomised to the control-delayed arm. In both groups, self-reported data will be collected at baseline (pregnancy) and when children are 24- and 36-months; and child clinical oral health status will be determined during standardised examinations conducted at 24- and 36-months by two calibrated dental professionals.

**Discussion:**

Expected outcomes will address whether exposure to a culturally-appropriate ECC intervention is effective in reducing dental disease burden and oral health inequalities among Indigenous children living in South Australia.

## Background

### Early childhood caries (ECC)

Early childhood caries is the presence of one or more decayed, missing, or filled primary teeth in children aged 71 months (5 years) or younger [1]. In children younger than 3 years, any sign of dental decay is considered Severe ECC, or S-ECC [2]. Early childhood caries not only causes pain, but also impacts children’s ability to eat, play and sleep [3]. It is associated with chronic childhood conditions and nutritional disorders [4], and is the strongest predictor of poor oral health later in childhood and into adulthood [5], when it is associated with clinical symptoms as well as psycho-social outcomes such as low self-confidence and poor perceived social acceptability.

### Indigenous Australian ECC disparities

Indigenous Australians are disadvantaged on almost every health and social indicator compared to non-Indigenous Australians [6]. Marked oral health disparities exist between Indigenous and non-Indigenous children in Australia; in some areas Indigenous children have up to five times the prevalence of early childhood caries as non-Indigenous children [7]. In light of how many chronic health conditions Indigenous Australians experience [8], oral health may not seem a priority. Yet oral health has important implications for general health and, crucially, there are interventions of known efficacy for preventing this avoidable disease [9]. If the burden of early childhood caries and associated oral health inequalities experienced by Indigenous children are to be reduced, more needs to be done to ensure that appropriate preventive measures, together with support for maintaining optimal oral health, are provided to carers of Indigenous children in the early life stages.

### ECC intervention rationale

The intervention undertaken in this study is comprised of four approaches reported to successfully prevent early childhood caries: dental care provided during pregnancy [10,11]; fluoride varnish applied to the teeth of children [12-15]; motivational interviewing [16-18]; and anticipatory guidance [19-22]. Most dental care during pregnancy is safe, and may aid in preventing early childhood caries because maternal transmission is the primary way in which children are inoculated with the bacteria, mutans streptococci, associated with early childhood caries. Fluoride varnish prolongs contact between fluoride and enamel and is particularly efficacious in preventing early childhood caries in Indigenous populations [12-15]. Reports indicate little difficulty with compliance and no adverse events; and non-dental personnel can be trained to apply fluoride varnish. Motivational interviewing (MI) is a non-judgmental, client-centred method of engaging intrinsic motivation to change behaviour by developing discrepancy and exploring ambivalence, in this case, about oral health. Employing strategies to move carers from inaction to action, motivational interviewing offers many possible paths to solutions [23]. Anticipatory guidance is a developmentally-based counselling technique that focuses on the needs of a child at particular stages of life. It has been effectively used in the dental setting to reduce ECC among high risk populations [22].

### Aim and hypotheses

This study seeks to determine if implementing a culturally-appropriate early childhood caries (ECC) intervention reduces dental disease burden and oral health inequalities among Indigenous children living in South Australia, Australia. We hypothesize that exposure to a culturally-appropriate ECC intervention will reduce dental disease burden among Indigenous children and reduce oral health inequalities between such children with their non-Indigenous counterparts. The South Australian study described in this paper is part of a larger investigation including research teams in New Zealand and Canada; we will compare findings when they become available.

## Methods

### Intervention review

**Figure 1:**
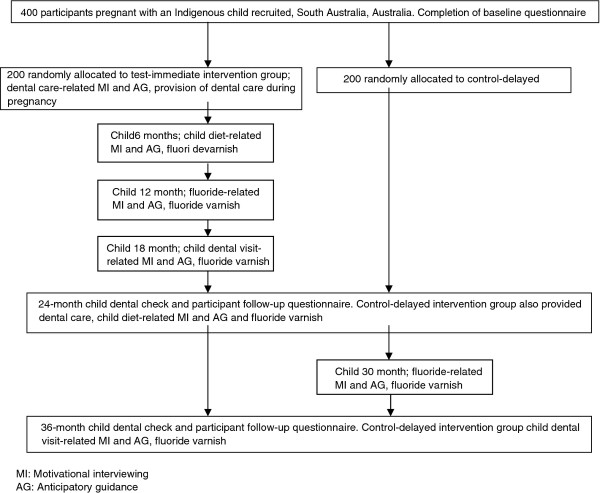
Study design schema.

In this randomised, controlled trial participants are offered four intervention components: (1) provision of dental care; (2) fluoride varnish application to the teeth of children; (3) motivational interviewing and (4) anticipatory guidance. A schema outlining the study design is presented in Figure 1.

1. Provision of dental careIn the test-immediate intervention group, dental care is arranged during pregnancy or up to six weeks after birth. For participants in the control-delayed intervention group, dental care is arranged when their child is 24 months of age. Care will comprise of extractions, restorations, scaling and prophylaxis and will take as many visits as required to achieve a non-diseased mouth. Participants holding an Australian healthcare or pensioner concession card will be eligible for treatment through the South Australian Dental Service; participants who do not hold this entitlement will receive treatment from private dentists limited to $1265 AUD. Dentists are asked to focus on reducing bacterial load, such as filling or extracting teeth with frank carious lesions. Study staff remind participants of dental appointments by telephoning, sending text messages via mobile phone and sending letters, and attempt to mitigate obstacles such as dental fear and lack of childcare by attending appointments when helpful. Free transport to and from the dentist is also available through culturally-sensitive transport providers.

2. Fluoride varnish applicationsFluoride varnish is applied to children by trained study staff in a setting convenient to participants, often their homes. In the test-immediate intervention group, this occurs when children are aged 6-, 12- and 18-months. Children in the control-delayed intervention group receive fluoride varnish applications at 24-, 30- and 36 months. Given the obstacles in meeting participants, these age targets are extended by one month prior and three months following target age, e.g. the six-month meeting can occur anywhere from five to eight months of age.

3. Motivational interviewingParticipants in the test-immediate intervention group receive four motivational interviewing (MI) sessions by trained study staff, with each session focussing on a specific directive. For participants in the test-immediate intervention group, the MI sessions occur at baseline (pregnancy) and when the child is aged 6-, 12- and 18-months. The directives for each of these sessions are: (1) Baseline/pregnancy; encourage participants to attend dental appointments; (2) Child aged 6-months; encourage participants to learn more about the importance of non-cariogenic foods and drinks for their child; (3) Child aged 12-months; emphasise to participants the importance of fluoride in early childhood caries prevention; and 4) Child aged 18-months; encourage participants to enrol their child for a dental check. Participants in the control-delayed intervention group receive three motivational interviews when the child is aged 24-, 30-, and 36 months, with the first session combining directives one and two.

4. Anticipatory guidanceAnticipatory guidance (AG) for those in the test-immediate intervention group will occur during pregnancy and when the child is aged 6-, 12-, and 18-months. Participants in the control-delayed-intervention group will receive anticipatory guidance when the child is aged at 24-, 30-, and 36 months. The anticipatory guidance topics reflect and compliment the directives of the MI sessions.

#### Participants and recruitment

Participants must be pregnant residents of South Australia expecting an Indigenous Australian baby or babies, or who have recently birthed an Indigenous Australian baby or babies less than six weeks of age. Although interventions are timed differently (the test-immediate intervention begins in pregnancy, while the control-delayed intervention begins when the child is aged 24- months), all participants are eligible to receive free dental care including transportation, fluoride varnish for children’s teeth, dental-related sample bags as well as gift cards valued at fifty dollars as reimbursement for time spent completing each questionnaire at baseline, 24- and 36- months. Participants are recruited through referrals from a variety of sources including Indigenous groups, community services and hospitals. Recruitment commenced January 31, 2011 and will end May 4, 2012.

#### Ethics and consent

This study received approval from the University of Adelaide Human Research Ethics Committee, the Aboriginal Health Council of South Australia, the Government of South Australia, the Human Research Ethics Committee of Child, Youth, and Women’s Health Service, and the Human Research Ethics Committees of participating Adelaide hospitals (i.e. Flinders Medical Centre, Lyell McEwin Hospital and Women’s and Children’s Hospital). Researchers receive guidance from an Indigenous reference group and heed the World Health Organisation principles that health research among Indigenous populations needs to be designed and carried out in a manner that takes account of cultural differences based on mutual respect, and is beneficial to both parties [24]. The study also takes into account local Indigenous South Australians’ principles that the project be sustainable, pro-active, and have an Indigenous community and family approach. Researchers are respectful of participants’ time and needs, and link families to other services whenever possible.

Potential participants are provided information about the study face-to-face and/or over the phone from health service providers and study staff. If potential participants consent to meeting, researchers explain the project in detail, answering any questions which arise. Those who want to participate are then asked to complete and sign a form expressing consent for various elements of the study including participation, access to child’s dental records, access to child’s hospital records and audio recording for the purposes of assessing researchers’ proficiency in motivational interviewing. Consent is obtained using the NHMRC Guidelines for Ethical Conduct in Aboriginal and Torres Strait Islander Health Research [25]. All participants are informed that their participation is voluntary and they can refuse or withdraw from participating at any stage without reason or justification. Participants receive a form explaining how to discuss their rights as a participant, raise concerns about the study, and/or make a complaint. Study results will be sent to interested participants.

#### Staff

Four staff have been employed for the Australian arm of the study; one Indigenous and three non-Indigenous.

#### Funding

The Australian arm of this international collaborative grant is provided by the National Health and Medical Research Council of Australia (NHMRC ICIHRP Grant application ID # 627350).

### Data collection

Clinical and self-reported data collected during interventions are entered into password-protected Microsoft Excel and Access databases at the Australian Research Centre for Population Oral Health, University of Adelaide.

#### Sample size

Based on a recent ECC intervention study of Australian Northern Territory-dwelling Indigenous children, it was estimated that a sample size of 250 (125 in each arm of the trial) would be necessary to detect a 25 percent difference in ECC prevalence between the two groups, at the significance criterion of 0.05 and a power of 0.80 [15]. The literature indicates that it is reasonable to expect a difference in effect of this magnitude following a motivational interviewing intervention among Indigenous children [16]. Allowing for an attrition rate of 35 percent after 36 months, 385 participants would be necessary at base-line; rounded up to 400 for convenience (200 test-immediate intervention group, 200 control-delayed intervention group).

#### Randomisation

Participants are randomly assigned on a 1:1 basis to either a test-immediate or control-delayed intervention group. A computer-generated permuted block randomisation sequence is used, stratified by six recruitment sites, i.e. Women’s and Children’s hospital, Flinders Medical Centre and Southern metropolitan, Lyell McEwin and Northern metropolitan, Whyalla and Port Pirie, Port Augusta, and non-metro regions including Murray Bridge, Mount Gambier, Port Lincoln and Ceduna. The randomisation was developed by biostatisticians at the Australian Research Centre for Population Oral Health (ARCPOH) using a random number generator. Randomly selected block sizes of 4, 6 and 8 were used, such that there is an equal number of participants in each intervention arm within the blocks. This ensures that if the study is stopped at any particular time there will be approximately an equal number of participants in each intervention arm.

#### Questionnaires

Self-report information in the form of a questionnaire will be administered by staff at baseline (pregnancy) and when children are aged 24- and 36-months. The questionnaire was pilot-tested using focus groups comprised of members of participating Indigenous communities and Aboriginal Maternal Infant Care workers, with the questions revisited and refined during Year 1 of the research. Questionnaire items were modelled on those used in both Australian [26] and international population-level surveys [27,28] and include carers’ self-reported ECC experience of their child, as well as carers’ oral health knowledge, oral self-care, dental service utilisation, oral health-related self-efficacy and oral health literacy.

Covariates include socio-demography, oral health and general health. Socio-demographic covariates (collected at baseline only) include age, Indigenous affiliation, residential location, education level, employment, gross household income, house ownership, number of children, number of people who stayed in house the previous night, and car ownership. Oral health covariates include carer self-reported oral health, oral health-related quality of life, and history and experience of dental services of both the parent and child. General health covariates include parent behaviours such as tobacco smoking, alcohol consumption and self-reported general health.

#### Clinical examinations

Information about child clinical oral health status will be collected during standardised examinations conducted at the 24- and 36-months follow-ups by two calibrated dental professionals. Before examination, a new toothbrush will be used to clean the teeth, and the teeth dried with gauze. Examiners will follow a standardised protocol. Procedures appropriate for young children will be used during the examinations, for example, children examined in the ‘knee-to-knee’ position on their carers’ lap. Standard infection control procedures will be followed with a fibre-optic light used as a light source. No sharp instruments will be used during examinations. The status of all teeth and tooth surfaces will be examined and recorded. Non-cavitated and cavitated lesions will be recorded for all teeth. The study will focus on the earliest manifestation of ECC. Two ECC case definitions will be used: (1) one or more upper incisor teeth labial surfaces being carious, either non-cavitated or cavitated and; (2) one or more non-cavitated or cavitated carious, missing of filled surfaces. Lesions will be diagnosed as ‘non-cavitated’ if an area of demineralization is without loss of surface continuity detected visually. Lesions will be diagnosed as ‘cavitated’ if a loss of continuity of the enamel is detected [2]. In addition, levels of tooth loss and dmft/s indices will be assessed from the tooth- and surface-level recorded observations. Diagnosis will be based on visual criteria only.

### Statistical Analysis

Test-immediate and control-delayed intervention groups will be compared at baseline, 24- and 36- months. Results will be compared using the Generalized Linear Mixed Model (GLMM) approach to focus on variation in the primary outcome: early childhood caries levels. All participants who completed the 24-month follow-up ECC-related outcome data will be included in multivariate modelling. Analysis will include fitting GLMM models for analysis of the correlated data in order to determine factors associated with ECC-related changes. Missing data will be addressed by the GLMM estimating mechanism, which assumes interdependence between repeated measures of participants, copes well with random missing data, allows for different intercepts and rates of change, and provides good predictive models. All variables showing associations at p < 0.15 in the GLMM univariate analyses will be entered into multivariate models using a stepwise approach. All effects will be estimated with 95% confidence intervals, with statistical significance taken as a two-sided p-value less than 0.05. This data will contribute to the inter-country analysis.

## Discussion

This study could provide a foundation for preventive dental care and oral health education among Indigenous children, reducing oral disease in childhood. The unique approach of combining four interventions of known efficacy in preventing or reducing early childhood caries will potentially enable greater benefits. The control-delayed intervention design satisfies many of the ethical concerns raised, in Australia at least, when conducting traditional randomised controlled trials among Indigenous populations (in which the control arm typically receive ‘standard course-of-care’ or no benefits at all) [25,29].

This study is timely, as most countries recognize the oral health of Indigenous children to be an important yet often over-looked area of both research and policy. Because each country participating in the study (i.e. Australia, New Zealand and Canada) has unique Indigenous population groups in terms of history and geographic isolation, our data will enable country, community and individual-level comparisons. The governments of Australia, New Zealand and Canada recognize Indigenous child oral health to be a priority in their respective national oral health plans; and Indigenous groups in these nations emphasise that children’s oral health is of key importance. Developing culturally-appropriate interventions which utilise Indigenous frameworks and research methodologies may provide evidence which leads to more appropriate oral health initiatives for Indigenous children, reducing global oral health inequalities.

## Abbreviations

AG, Anticipatory guidance; dmft/s, Decayed, missing or filled teeth/surfaces in the primary dentition; ECC, Early childhood caries; GLMM, Generalized linear mixed model; MI, Motivational interviewing; MS, Mutans streptococci; NHMRC, National Health and Medical Research Council of Australia.

## Competing interests

The authors declare that they have no competing interests.

## Authors’ contributions

JM participated in data collection, data management and manuscript preparation. AC, EP, KRT, GM, AJS, JB and HL provided important intellectual input into the study design and revision of the manuscript. LJ participated in study design, ethics applications, coordinated data collection and data management, and participated in manuscript preparation. All authors were involved in revising the manuscript for important intellectual content and read and approved the final manuscript.

## Pre-publication history

The pre-publication history for this paper can be accessed here:

http://www.biomedcentral.com/1471-2458/12/323/prepub
